# Correlation between Clinical and Immunological Variables and Humoral Response to SARS-CoV-2 Vaccination in Adult Patients with Antibody Deficiency Disorders

**DOI:** 10.3390/pathogens11111364

**Published:** 2022-11-16

**Authors:** Carmen Bracke, Cristina Miranda, Sandra González, Irma Casas, Pere Joan Cardona, Rosa Maria Benitez, Nieves Sopena, Esteban Alberto Reynaga, Marta Massanella, Bonaventura Clotet, Jorge Carrillo, Lourdes Mateu, Maria Luisa Pedro-Botet

**Affiliations:** 1 Department of Infectious Diseases, Germans Trias i Pujol Hospital, 08916 Badalona, Spain; 2 Fight AIDS and Infectious Diseases Foundation, 08916 Badalona, Spain; 3 Department of Preventive Medicine, Germans Trias i Pujol Hospital, 08916 Badalona, Spain; 4 Microbiology Department, Germans Trias i Pujol Hospital, 08916 Badalona, Spain; 5 Department of Genetics and Microbiology, Autonomous University of Barcelona, 08193 Cerdanyola, Spain; 6 Respiratory Disease Networking Biomedical Research Center (CIBERes), Carlos III Health Institute, 28029 Madrid, Spain; 7 IrsiCaixa AIDS Research Institute, Germans Trias i Pujol Hospital, 08916 Badalona, Spain; 8 Infectious Disease Networking Biomedical Research Center (CIBERINFEC), Carlos III Health Institute, 28029 Madrid, Spain

**Keywords:** COVID-19 vaccination, antibody deficiency disorders, primary immunodeficiencies, secondary immunodeficiencies, COVID-19 vaccination response, GLILD, immunosuppressive therapy

## Abstract

Background. Prophylactic vaccination has proven to be the most effective strategy to fight the COVID-19 pandemic. Methods. This was a prospective observational cohort study involving 30 predominantly antibody deficiency disorders (ADD)-afflicted adult patients on immunoglobulin replacement therapy vaccinated with three doses of the mRNA-1273 COVID-19 vaccine, and 10 healthy controls. Anti-RBD IgG antibodies were determined in plasma samples collected just before the first dose of mRNA-based COVID-19 vaccine and on weeks 4, 8, 24, and 28 following the first vaccination. Patients were categorized based on the levels of anti-RBD antibodies determined on w8 as non-, low-, and responders. Chi-square and Kruskal–Wallis tests were used to see if any variables correlated with humoral response levels. Any adverse effects of the mRNA-based vaccine were also noted. Results. The COVID-19 vaccine was safe and well-tolerated. The humoral response elicited at w8 after vaccination depended on the type of ADD, the type of immunoglobulin deficiency, the presence of granulomatous lymphocytic interstitial lung disease, recent use of immunosuppressive drugs, and the switched memory B cells counts. The third vaccine dose boosted humoral response in previous responders to second dose but seldom in non-responders. Conclusions: The humoral response of patients with predominant ADD depends mostly on the type of immunodeficiency and on the frequency of B and T cell populations.

## 1. Introduction

The first case of severe acute respiratory syndrome coronavirus 2 (SARS-CoV-2) causing coronavirus-induced disease (COVID-19) was reported in Wuhan, China, in late 2019. Since then, the virus has spread worldwide and more than 598 million cases have been identified, causing more than 6.4 million deaths as of August 2022 [1].

The development of different vaccines that have proven to be safe and effective [2,3] has dramatically reduced hospitalization, morbidity, and mortality associated with COVID-19. With this goal, a massive vaccination campaign was initiated in Spain in late December 2020 and continues to date, prioritizing the elderly, healthcare workers, and patients with chronic diseases.

Since the beginning of the pandemic, various studies have been conducted in populations affected by primary or secondary immunodeficiencies (PID and SID, respectively), with interesting results. Before the availability of COVID-19 vaccines, different observational studies in populations with inborn errors of immunity (IEI) suggested that this group of patients has a higher risk of mild to severe infection than the general population, as well as a higher case-fatality ratio [4]. Other studies, by contrast, reported that risk factors and mortality were comparable to those of the general population [5]. Such different conclusions may indicate that not all IEIs have either the same cumulative incidence or the same infection-fatality rate. In fact, in an Italian cohort, conditions associated with T-cell defects such as common variable immunodeficiency (CVID), Good syndrome, and Del 22q11 were reported to have the poorest figures [6]. Along the same lines, Ku et al. [7] suggested that the presence of autoantibodies to interferons can lead to uncontrolled inflammation and severe COVID-19 disease in patients with IEI. Moreover, chronic lung disease related to IEI such as bronchiectasis or granulomatous lymphocytic interstitial lung disease (GLILD) seemed to be risk factors for severe disease and mortality [8]. Since bronchiectasis and GLILD are well-known complications of CVID, this may explain why the rates of hospitalization and mortality in patients with COVID-19 have been shown to be higher in a subgroup of patients with CVID.

Concerning patients with SID, a study showed that the case-fatality ratio of COVID-19 was higher than for both the general population and patients with PID, though it should be noted that most SIDs in the study were related to hematological malignancies, a well-known independent risk factor for COVID-19 morbidity and mortality [9]. Thus, it is uncertain whether the immunodeficiency itself could be a risk factor for severe COVID-19 disease.

Since patients with antibody deficiency disorders (ADD) with COVID-19 seem to have poorer prognoses, prophylactic vaccination might be the most effective preventive strategy [10]. Studies of immunogenicity of COVID-19 vaccines in IEI have been conducted worldwide, yielding heterogeneous results. A few of these studies have evaluated the effects of the humoral response after a third dose of COVID-19 vaccine. However, most of the recently published multicenter studies have involved patients who were vaccinated with different types of vaccines. Furthermore, these patients represented the full spectrum of IEI rather than only those in need of immunoglobulin replacement therapy (IRT). Finally, it should be noted that very few studies have attempted to detect any relationships between clinical or analytical variables and the response to the vaccine in patients with ADD.

We recently evaluated the cellular and humoral responses developed in ADD individuals after three mRNA-1273 vaccine doses (10.21203/rs.3.rs-1551351/v1) [11]. The results showed that the vaccine-induced immune responses were highly heterogeneous among individuals. However, at the moment we have a poor understanding of the factors that can play a role in impairing the response to vaccination. To partly address this gap in our knowledge, in the present study we investigate whether particular patient characteristics could be related to the lack of humoral response in these individuals.

## 2. Patients and Methods

### 2.1. Study Design

This was a prospective observational efficacy cohort-comparative single-center study conducted at the Hospital Universitari Germans Trias i Pujol (HUGTiP) located in Badalona, Spain. The HUGTiP is a 650-bed tertiary referral hospital that serves the 800,000 inhabitants of the north metropolitan area of Barcelona. The study’s goal was to elucidate which factors have an impact on vaccine-specific immune responses after three doses of mRNA-based COVID-19 vaccine in PID and SID patients on IRT. The study, which received prior approval from the institutional Board of Ethics (code: PI-21-107) began in April 2021 and ended in November 2021.

### 2.2. ADD Patients and Healthy Controls

Patients on IRT older than 18 years of age were selected from a database registry, maintained by the HUGTiP’s Infectious Diseases Department since 1986, of PID and SID patients who were clinically and analytically stable. Underlying IEIs in the PID population were defined according to the 2019 European Society for Immunodeficiencies (ESID) criteria [12].

Patients with any of the following conditions were excluded: previous COVID-19 vaccination, vaccine-induced anaphylactic reaction, documented allergy to polysorbate or polyethylene glycol, and pregnant or breastfeeding women.

Healthy controls who were age- and gender-balanced from the King cohort extension (HUGTiP/PI-20-217) and who had received two doses of mRNA-based COVID-19 vaccine were included for the purpose of comparing humoral responses to vaccination.

Prior written informed consent was obtained from all participants.

### 2.3. Method

The primary endpoint was to categorize humoral responses in terms of antibody levels against Receptor Binding Domain (RBD) in ADD patients compared with healthy controls after two doses of COVID-19 vaccine.

Secondary endpoints were to evaluate whether humoral responses to two doses of vaccine were related to any of the basal clinical and analytical characteristics of the patients, to determine whether a third dose was able to booster humoral responses, and to describe adverse effects related to any of the mRNA-based vaccine doses.

All participants received two doses of the mRNA-based COVID-19 vaccine 28 days apart (April–May 2021) as described in (10.21203/rs.3.rs-1551351/v1) [11]. Blood samples were collected just before the first dose (week 0) and at weeks 4 (following the second vaccination), 8, and 24. In September 2021, the Spanish Health Ministry updated recommendations regarding vaccination for COVID-19, and an additional third dose was administered to ADD patients at week 24. An extra blood sample was collected from these patients at week 28, four weeks after administration of the third dose.

Variables such as demographic data, underlying conditions, immunosuppressive agents received in the last three months, previous rituximab, ESID registry-based categorization of immunodeficiency, years since PID/SID diagnosis, type of immunoglobulin (Ig) deficiency in PID/SID, GLILD, isohemagglutinin levels, Typhim Vi^®^ and *S. pneumoniae* polysaccharide antibody response, type of IRT, years on IRT, last available IgG trough levels, previous COVID-19 infection, and lymphocyte subpopulation counts were collected from clinical charts in an SPSS database.

We performed telephonic clinical controls at weeks 4, 8, 24, and 28 and had participants complete a questionnaire reporting adverse effects after each dose of vaccine at weeks 5, 9, and 29.

### 2.4. Humoral Response to COVID-19 mRNA-Based Vaccine 

Anti-RBD IgG antibodies in plasma samples were determined as described in (10.21203/rs.3.rs-1551351/v1) [11]. In brief, plasma samples were heat-inactivated and assayed in duplicate against the RBD protein (Sino Biological, Beijing, China) or antigen-free wells. A serially-diluted positive plasma sample was used as standard, and a pool of ten SARS-CoV-2-negative plasma samples were included as negative controls. Samples were incubated for two hours at room temperature. A horseradish peroxidase (HRP)-conjugated goat anti-human IgG (Fc-specific) (Jackson Immunoresearch, West Grove, PA, USA) was used as secondary antibody and O-phenylenediamine dihydrochloride (OPD) was used as a substrate (Sigma Aldrich, St. Louis, MO, USA). The enzymatic reaction of HRP was stopped with 2 M of H_2_SO_4_ (Sigma Aldrich). Signal was analyzed as the optical density at 492 nm with noise correction at 620 nm. Antigen-specific signal was calculated by subtracting background signal obtained for each sample in antigen-free wells. Results are shown as arbitrary units (AU)/mL.

### 2.5. Statistical Analysis

Chi-square or Fisher’s exact tests were performed to compare categorical variables from ADD patients classified according to their humoral response against the COVID-19 vaccine into three groups: non-responders (NR), low responders (LR), and responders (R).

The Kruskal–Wallis test was used to compare numerical variables with levels of humoral response to vaccination. The differences were considered statistically significant when a *p*-value was <0.05. Analyses were performed using IBM SPSS Statistics for Windows, version 24 (IBM Corp., Armonk, NY, USA).

## 3. Results

Thirty patients with ADD on IRT and 10 healthy controls were included in the study.

### 3.1. SID and PID Patients’ Characteristics

The main characteristics of ADD patients are shown in Table 1. Only three patients had SID (3/30; 10%) and, in accordance with the ESID criteria, PID patients were classified into the following groups: CVID (14/30; 46.7%); unclassified predominantly antibody deficiency (unPAD) (11/30; 36.7%); combined immunodeficiency (CID) (1/30; 3.3%); and thymoma and immunodeficiency (TI) (1/30; 3.3%). Five out of 30 patients (5/30; 16.7%) were or had been on immunosuppressive drugs in the three months prior to inclusion in the study, with one of them having received Rituximab one month prior to the first dose of the vaccine. The immunosuppressive treatment administered was azathioprine in two patients, mycophenolate in one patient, a high dose of corticosteroids in one patient, and a combination of rituximab and corticosteroids in one patient. This treatment was administered because of GLILD in three patients (azathioprine, corticoids and rituximab, respectively) and in the other two patients because of an underlying disease causing SID (vasculitis and nephrotic syndrome, receiving azathioprine and mycophenolate respectively). Four patients (4/30; 13.3%) had been previously diagnosed with COVID-19 infection, with two (2/4; 50%) requiring hospitalization because of low oxygen levels.

### 3.2. Adverse Reactions to Vaccination

Local pain was the most frequently reported adverse effect of vaccination (83.3%, 90% and 83.3% after the first, second, and third doses, respectively). No severe adverse effects were reported. Only one patient had to go on sick leave after the first and second dose, due to fever and vomiting, but did not require medical assistance or hospitalization. All adverse effects are summarized in Table 2.

### 3.3. Two-Dose Vaccine-Induced IgG Immune Response Categorization and Related Variables

All healthy controls yielded Spike-specific IgG titers at week 8. By contrast, patients with ADD showed heterogeneous humoral responses after two doses of mRNA-based vaccine.

**Figure 1:**
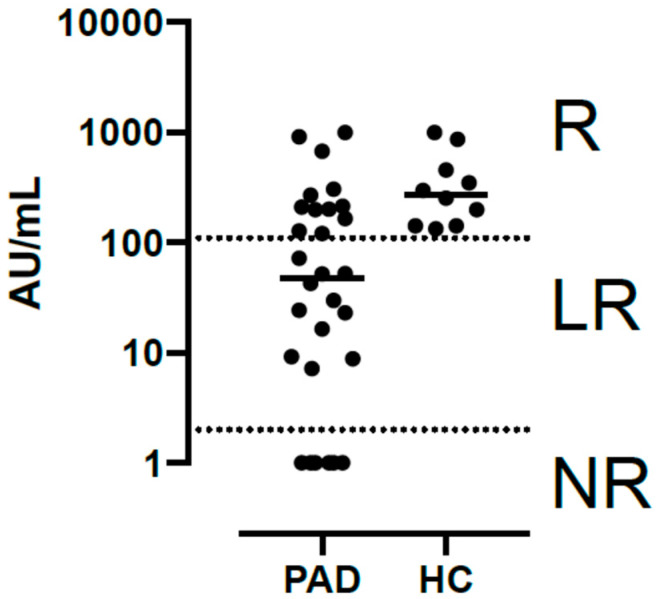
Antibody levels in predominantly antibody deficient disorders patients and healthy controls at week 8 categorized as non-responders (NR), low responders (LR) and responders (R).

After comparing antibody levels in ADD patients and healthy controls at week 8, we classified SID and PID patients into three groups according to the level of their response: those whose antibody levels were undetectable were labeled ‘non-responders’ (NR) (7/30; 23.3%); those who had lower titers than healthy controls were labeled ‘low responders’ (LR) (11/30; 36.7%), and those who had titers comparable to those of healthy controls were labeled ‘responders’ (R) (12/30; 40%) (Figure 1).

No statistically significant differences were observed between sex, age, underlying or IEI-related diseases, previous rituximab, IgG trough levels, isohemagglutinin levels or response to the polysaccharide vaccine against *Streptococcus pneumoniae* or *Salmonella typhi*, and humoral response to two doses of COVID-19 vaccine. Although not significantly different, mean absolute B lymphocyte count and mean absolute CD4 positive lymphocyte count tended to be higher in R patients than in LR or NR, and mean percentage of transitional B lymphocytes tended to be lower in R than in LR and much lower than NR (Table 3). By contrast, as shown in Table 4, the type of immunodeficiency as per the ESID registry, the type of Ig deficiency, the presence of GLILD, having received immunosuppressive drugs in the last three months, and mean percentage of switched B lymphocytes were significantly related to the level of anti-RBD IgG antibodies after the second dose of the COVID-19 vaccine. With regard to type of immunodeficiency, all three patients with SID (3/3; 100%) and most unPAD individuals (7/11; 63.6%) were R, while the one patient affected by TI was NR and most patients in the CVID group were either LR (7/14; 50%) or NR (5/14; 35.7%) (*p* = 0.005). Concerning the type of Ig deficiency, 2 out of 16 patients (12.5%) with IgG, IgA, and IgM deficiency were R, while 5 out of 6 (83.3%) with IgG deficiency only were R. In the same line, 7 out of 16 (43.8%) with IgG, IgA, and IgM deficiency were NR, while none of the 6 patients with IgG deficiency only were NR (*p* = 0.02). Interestingly, all patients with GLILD were NR (3/3; 100% vs. 12/27; 44.4%) (*p* = 0.004). Patients who had received immunosuppressive drugs in the last three months were more frequently NR (3/5; 60% vs. 4/25; 16%) (*p* = 0.05). Finally, mean percentage of switched memory B cells was significantly higher in LR and R patients (15.46 and 11.8, respectively) than in NR patients (1.74) (*p* = 0.05).

### 3.4. Third-Dose Vaccine-Induced IgG Immune Response against SARS-CoV-2

After an additional third dose was administered to ADD patients at week 24, a booster effect was observed at week 28 in 19 out of 29 patients (one R patient refused to take a blood test at week 28): one NR (1/7; 14.3%), seven LR (7/11; 63.6%), and all R (11/11; 100%).

A descriptive analysis of the 10 patients (10/29; 34.5%) who did not experience a booster effect after the third vaccine dose is outlined below. Six of them were females, and the mean age was 52.5 years (29–71). Eight were diagnosed with CVID, one was diagnosed with TI, and another (10%) was diagnosed with unPAD. Six of the 10 showing no booster effect were previously NR and four were LR. All three patients diagnosed with GLILD were NR after two doses and did not show a booster effect after the third dose of the vaccine. These three patients with GLILD had been on immunosuppressive drugs during the three months prior to the third vaccination (three patients receiving rituximab, a high dose of corticoids, and azathioprine, respectively). Four of the 10 showing no booster effect had received rituximab in the past, three of them because of GLILD and the other due to a lymphoma. Mean levels of B lymphocytes were 87.5 cel/µL (0–215).

## 4. Discussion

In this study, we analyzed humoral response in 30 ADD patients (CVID, unPAD, TI, CID or SID) after they had received three doses of an mRNA-based COVID-19 vaccine. The results of this analysis provide information that is crucial in giving our best assessment to ADD patients, who are a high-risk group of individuals, in the context of a global infectious pandemic.

Although some similar research has recently been published [14,15], the aim of our study was to analyze whether any relationship could be identified between clinical or analytical patient variables and immune responses in order to better manage and inform patients with ADD in terms of minimizing the risk of COVID-19 infection. In these previous publications, the range of humoral response varied from 20% to 80%, and it has been suggested that low B cell levels [16,17] or immunosuppressive treatment [18] such as B cell depletion therapy [19] predict a weak serological response. However, other studies did not find any relation between these factors and poorer humoral and/or cellular response. Nevertheless, these studies exhibit certain limitations, such as the heterogeneity observed in immunological impairment and the use of different vaccine schemes.

In our study we only included ADD patients on IRT, which implies a more advanced stage of the disease, sometimes involving recurrent infections and/or lung damage. This might be a factor underlying an inadequate vaccine response, given that the immune systems of this population are more impaired. On the other hand, studies in this area have been performed involving patients with different vaccine schemes including heterologous vaccination, which is already known to be more immunogenic than the homologous scheme [20]. We consider that homogeneity in the severity of IEI as well as in vaccination schemes may help to avoid the presence of confusing factors.

A phase 3 randomized, observer-blinded, placebo-controlled trial carried out in the United States evaluated the safety of the mRNA-1273 vaccine. A comparison of those who received two doses of mRNA-1273, 100ug each, with those who received placebo showed that local and systemic adverse events occurred more frequently in the mRNA-1273 group, especially after the second dose and among younger people (18 to <65 years of age). Pain was the most frequent injection-site event, while fever, headache, fatigue, myalgia, arthralgia, nausea or vomiting, and chills were the most frequent systemic adverse events. It should be noted that severity of systemic adverse events was higher after the second dose [2]. Since that publication, some other local and systemic reactions have been described after the administration of both mRNA BioNTech-Pfizer BNT162b2 and mRNA-1273 (Moderna) vaccines [21,22]. The results of our own study show that mRNA-based COVID-19 vaccines are safe and well tolerated in patients with ADD. Here, we have shown that the humoral response to the vaccine in such patients depends on the type of ADD and, in consequence, on the type of Ig deficiency. In addition, the use of immunosuppressive drugs, the presence of GLILD, and low percentages of switched memory B cell all seem to be related to a lack of immune response to two doses of COVID-19 vaccine. An additional third booster does increase humoral response in patients who previously had a high response to the second dose, but this is rarely the case in those who did not respond to the second dose.

It seems logical to suppose that COVID-19 vaccine response will be different depending on the ESID registry-based type of ADD, which mainly takes into account the specific immune impairment. However, the studies currently published on COVID-19 vaccine response in patients with IEI distinguish SID from PID patients and the different ESID registry-based levels of PID from each other from the outset. By contrast, the study design employed here has us allowed to demonstrate that the immune response correlates with the type of immunodeficiency and, in consequence, with the type of Ig deficiency. Specifically, we observed that, compared to healthy controls, patients with TI (1/1 NR) and CID (1/1 NR) had the poorest humoral response after two doses of vaccine, patients with CVID had heterogeneous responses, although overall they had poor responses (5/14 NR, 7/14 LR, 2/14 R), and that most unPAD patients (7/11; 63.6%) and all patients with SID (3/3; 100%) had a good humoral response. As in another studies [18], our work shows that the use of immunosuppressive drugs three months before vaccination was related to a poorer response to the vaccine. In our cohort, one patient who had received rituximab proved to be NR. As several other studies have demonstrated [23], B-cell depletive treatment reduces antibody responses for at least six months after the last dose administered, thus correlating to a NR level of response. In a study conducted in patients with autoimmune inflammatory rheumatic diseases [24], one patient from the cohort had been receiving high doses of glucocorticoids, a treatment also linked to poorer humoral response, and also had no detectable response to vaccination. Two other patients in our study were on azathioprine; in this case, one was NR and the other was R. The explanation may be that the patient who was NR had CVID and was receiving 100mg/day of azathioprine, while the R patient was SID due to a vasculitis and was receiving 50mg/day of azathioprine; as noted above, CVID seems to be associated with poorer response, whereas SID correlates with R. The last patient described in the study was on mycophenolate, which has also been linked to poorer vaccine response [24], yet this patient had high antibody titers. It is important to point out that this patient was also SID due to nephrotic syndrome, so perhaps the type of immunodeficiency disorder plays a stronger role in the humoral response than the type of immunosuppressive treatment. This may be due in part to the type of Ig deficiency, which significantly correlated with the response to the vaccine in our study. In fact, patients with IgG deficiency only were R significantly more frequently than those who had an IgG and IgM deficiency, more than those who had IgG and IgA or than those who combined IgG, IgA, and IgM deficiency such as CVID, TI, and CID.

With regard to the patients in our study affected with GLILD, they all proved to be NR (*p* = 0.004). It is well known that GLILD is a non-infectious complication of CVID patients, where lymphocytic granulomas commonly affect the lungs, and its treatment includes high doses of glucocorticoids, rituximab, or mycophenolate. As the presence of GLILD involves being affected with CVID and being on immunosuppressive drugs, both factors related to poorer humoral response, this could be a confusing factor. It should be noted that all patients with GLILD included here were receiving immunosuppressive treatment throughout the study.

Although it was not statistically significant, we observed a tendency for the absolute B lymphocyte cell count, absolute count of T lymphocytes CD4+, and percentage of memory B cells to have higher values in the R group than in the LR or NR groups. In addition, the percentage of transitional B cells tended to be higher in the NR group than in the LR or R groups. It has already been suggested that lymphocyte subpopulation counts seem to be related to SARS-CoV-2 vaccine responses, especially in populations receiving B cell depletion therapy [25]. With regard to IEI, low peripheral CD19^+^ cell count has been suggested as a factor for inadequate vaccine response. Only a few studies have analyzed lymphocyte subpopulation counts, and the only correlation they identified was between low switched memory B cells and an inadequate response to vaccination in populations affected by CVID [23]. In our own results, patients with low switched memory B cells were more frequently NR. Since switched memory B cells are antigen-specific B cells, their absence suggests a problem in B cell differentiation [26,27], as in B cell depletion therapy or in CVID.

Although advanced age is known to be a risk factor for an inadequate response to COVID-19 vaccines in the general population [28], as in EIE [29], this was not observed in our study.

After a third dose of mRNA-based COVID-19 vaccine, we observed that the boost in humoral immunity was rare in those patients that were non-responders after the second dose (1/7NR).

The limitations of our study include a small sample size, the heterogeneity of the immunological impairments affecting the cohort, and the poor representation of some categories of IEI in this sample, especially for CID, TI and SID.

## 5. Conclusions

Based on the results of this study, we can conclude that the COVID-19 vaccine is safe in PID and SID patients, and that three different groups (non-responders, low responders and responders) can be identified when compared to humoral response in healthy controls. ESID-registry-based classification (CID, TI, and CVID), type of Ig deficiency, presence of GLILD, use of immunosuppressive drugs, and low percentage of switched B cells are all factors related to inadequate humoral response to mRNA-based COVID-19 vaccines. A third dose is not able to improve the humoral response in patients previously NR to two doses. Although these results should be confirmed in larger series, other preventive strategies such as tixagevimab/cilgavimab [30] or early administration of antiviral regimes [31,32] should therefore be considered in patients with CVID, CID, or TI. In contrast, based on the variability of their humoral response, unPAD patients should be evaluated with caution and other variables such as lymphocyte subpopulations counts should be taken into account. Finally, SID patients seem to behave as healthy controls.

Future research on humoral and cellular response in larger samples should be performed in order to more precisely identify factors that predict inadequate vaccine response.

## Figures and Tables

**Table 1:** Patient characteristics.

Variable	n/30 (%)
Age in years—mean (range)	55.6 (29–85)
Gender	Female 15 (50%)
	Male 15 (50%)
Underlying or related diseases	Lymphoma 2 (6.7%)
	Leukemia 1 (3.3%)
	Solid cancer 7 (23.2%)
	Basocellular carcinoma 2 (6.7%)
	Breast carcinoma 2 (6.7%)
	Lung adenocarcinoma 1 (3.3%)
	Seminoma 1 (3.3%)
	Thymoma 1 (3.3%)
	Chronic liver disease 1 (3.3%)
	Chronic kidney disease 1 (3.3%)
	Nephrotic syndrome 1 (3.3%)
	Autoimmune diseases (33.3%)
	Thrombotic thrombocytopenic purpura 2 (6.7%)
	Celiac disease 1 (3.3%)
	Collagenous colitis 1 (3.3%)
	Crohn’s disease 1 (3.3%)
	Ulcerative proctitis 1 (3.3%)
	Autoimmune anemia 1 (3.3%)
	Lupus-like syndrome 1 (3.3%)
	Membranoproliferative glomerulonephritis type 1 1 (3.3%)
	PR3-ANCA-associated vasculitis 1 (3.3%)
	GLILD 3 (10%)
	Asthma 6 (20%)
	Drug or food allergies 9 (30%)
Immunosuppressive agents used in the last 3 months	Azathioprine 2 (6.7%)
	Mycophenolate 1 (3.3%)
	Corticosteroids 1 (3.3%)
	Rituximab + corticosteroids 1 (3.3%)
Previous rituximab	6 (20%)
ESID registry for Immunodeficiency [12]	Combined Immunodeficiency 1 (3.3%)
	Common Variable Disorders 14 (46.7%)
	Secondary Immunodeficiency 3 (10%)
	Thymoma with Immunodeficiency 1 (3.3%)
	Unclassified Antibody Deficiency 11 (36.7%)
Years since diagnose diagnosis—mean (range)	7.87 (0–31)
Type of Immunoglobulin deficiency in PID/SID	IgG 6 (20%)
	IgG + IgA 4 (13.3%)
	IgG + IgA + IgM 16 (53.3%)
	IgG + IgM 4 (13.3%)
IgG subclass deficiency	IgG 1 4 (13.3%)
	IgG 1 + 2 + 3 1 (3.3%)
	IgG 1 + 2 + 3 + 4 6 (20%)
	IgG 1 + 2 + 4 13 (43.3%)
	No subclass affected 6 (20%)
Isohemagglutinin evaluation	Total evaluated 23 (76.7%)
	High rates 8 (26.7%)
	Low rates 13 (43.3%)
	Not evaluable ^1^ 2 (6.7%)
Polysaccharide Typhim Vi^®^ Antibody Response	Total evaluated 13/30 (43.3%)
	Adequate 6 (20%)
	Non-adequate 7 (23.3%)
Type of Immunoglobulin Replacement Therapy (IRT)	Subcutaneous 17 (56.6%)
	Intravenous 13 (43.4%)
Years in IRT	<1 year 5 (16.6%)
	1–5 years 13 (43.3%)
	5–10 years 6 (20%)
	>10 years 6 (20%)
Last available IgG trough levels—mean (range)	797 mg/dL (233–1112)
Previous COVID-19 infection	4 (13.3%)
WHO-SCORE in COVID-19 infection [13]	Score 1 1 (3.3%)
	Score 2 1 (3.3%)
	Score 5 2 (6.7%)
IgG SARS-CoV-2 (June/2020)	Positive 1 (3.3%)
	Negative 28 (93.3%)

^1^ Not applicable in the case of AB blood group.

**Table 2:** Adverse effects related to mRNA-based COVID-19 vaccine.

Adverse Events n/30 (%)	First Dose	Second Dose	Third Dose
Local Pain	25 (83.3%)	27 (90%)	25 (83.3%)
Local Blush	3 (10%)	5 (16.7%)	7 (23.3%)
Local Inflammation	3 (10%)	5 (16.7%)	8 (26.7%)
Paresthesia	0 (0%)	1 (3.3%)	2 (6.7%)
Headache	9 (30%)	8 (26.7%)	9 (30%)
Shivers	6(20%)	10 (33.3%)	12 (40%)
Arthromyalgia	7 (23.3%)	12 (40%)	12 (40%)
Asthenia	12 (40%)	15 (50%)	17 (56.7%)
Dizziness	1 (3.3%)	4 (13.3%)	4 (13.3%)
Syncope	0 (0%)	0 (0%)	0 (0%)
Nausea/Vomiting	1 (3.3%)	2 (6.6%)	1 (3.3%)
Diarrhea	2 (6.6%)	1 (3.3%)	3 (10%)
Fever	4 (13.3%)	10 (33.3%)	12 (40%)
Local Adenopathy	0 (0%)	0 (0%)	1 (3.3%)
Anaphylaxis	0 (0%)	0 (0%)	0 (0%)
Other Adverse Events	2 (6.6%)	5 (16.7%)	5 (16.7%)
Medical Assistance	0 (0%)	1 (3.3%)	0 (0%)
Sick Leave	1 (3.3%)	1 (3.3%)	0 (0%)
Days of Sick Leave	2	3	0

**Table 3:** Univariate analysis of lymphocyte B cells subpopulations related to second dose COVID-19 vaccine humoral response.

Variable	NR	LR	HR	*p* Value
Absolute B lymphocyte count (cel/µL)	102	137	262	>0.05
Mean Memory B Cells (%)	7.72	18.11	10.95	>0.05
Mean IgM memory B Cells (%)	1.42	2.10	1.6	>0.05
Mean Transitional B Cells (%)	12.7	9.15	8.25	>0.05
Mean Switched B Cells (%)	1.74	15.46	11.80	0.05
Mean absolute CD8 Count (cells/µL) (range)	561.6 (56–1068)	688 (265–1360)	350 (338–362)	>0.05
Mean absolute CD4 Count (cells/µL) (range)	578.2 (105–1022)	670.75 (274–1133)	1332 (1189–1476)	>0.05

**Table 4:** Significant related variables to second dose COVID-19 vaccine humoral response in a univariate analysis.

	Non-Responders n (%)	Low Responders n (%)	Responders n (%)	*p* (Ẋ^2^)
ESID registry classification				0.005
CID	1/1 (100)	0	0	
CVID	5/14 (35.7)	7/14 (50)	2/14 (14.3)	
SID	0	0	3/3 (100)	
TI	1/1 (100)	0	0	
UnPAD	0	4/11 (36.4)	7/11 (63.6)	
Type of Ig defciency				0.02
IgG	0/6	1/6 (16.7)	5/6 (83.3)	
IgG + IgM	0/4	1/4 (25)	3/4 (75)	
IgG + IgA	0/4	2/4 (50)	2/4 (50)	
IgG + IgA + IgM	7/16 (43.8)	7/16 (43.8)	2/16 (12.5)	
GLILD				0.004
YES	3/3 (100)	0	0	
NO	12/27 (44.4)	11/27 (40.7%)	4/27 (14.8)	
Immunosuppressive drugs < 3 months				0.05
YES	3/5 (60)	0	2/5 (40)	
NO	4/25 (16)	11/25 (44)	10/25 (40)	
Switched B cells (mean%, range)	1.74 (0–9)	15.46 (0–59)	11.80 (5–19)	0.05

CID: combined immunodeficiency; CVID: common variable immunodeficiency; SID: secondary immunodeficiency; TI: thymoma and immunodeficiency; UnPAD: unclassified predominantly antibody deficiency; Ig: immunoglobulin; GLILD: granulomatous lymphocytic infiltration lung disease.

## Data Availability

Not applicable.
